# Electrotaxis behavior of droplets composed of aqueous Belousov-Zhabotinsky solutions suspended in oil phase

**DOI:** 10.1038/s41598-023-27639-8

**Published:** 2023-01-24

**Authors:** Oliver Back, Munehiro Asally, Zuowei Wang, Yoshikatsu Hayashi

**Affiliations:** 1 Department of Biomedical Sciences and Biomedical Engineering, School of Biological Sciences, University of Reading, Reading, UK; 2 School of Life Sciences, University of Warwick, Coventry, UK; 3 Department of Mathematics and Statistics, School of Mathematical, Physical and Computational Sciences, University of Reading, Reading, UK

**Keywords:** Physical chemistry, Chemical physics, Thermodynamics

## Abstract

Taxis is ubiquitous in biological and physical chemistry systems as a response to various external stimulations. We prepared aqueous droplets containing Belousov-Zhabotinsky (BZ) solutions suspended on an oleic acid oil phase subject to DC electric field and found that these BZ droplets undergo chemically driven translational motion towards the negative electrode under DC electric field. This electrotaxis phenomenon originates from the field-induced inhomogeneous distribution of reactants, in particular Br$^{-}$ ions, and consequently the biased location of the leading centers towards the positive electrode. We define the ’leading center’ (LC) as a specific location within the droplet where the BZ chemical wave (target pattern) is initiated. The chemical wave generated from the LC propagates passing the droplet center of mass and creates a gradient of interfacial tension when reaching the droplet-oil interface on the other side, resulting in a momentum exchange between the droplet and oil phases which drives the droplet motion in the direction of the electric field. A greater electric field strength renders a more substantial electrotaxis effect.

## Introduction

Study of taxis phenomena has attracted a lot of interest in the fields of biology and physical chemistry, demonstrating the minimum or primitive form of intelligence. All living systems can sense external stimulations, process information, make an appropriate reaction, or show adaptive behavior.

Many biological systems exhibit taxis as a response to environmental changes^1,2^. For example, organisms like *E. coli* have demonstrated chemotaxis phenomenon by changing their moving trajectories in response to a detectable chemical gradient^3^. Similarly, the chemotaxis mechanism was utilized to guide immune cells to reach infection sites, allowing wounds to heal^4^. Magnetotaxis and electrotaxis phenomena were also observed in various bacteria. Magnetotactic bacteria can align their cell orientations with the magnetic field of the planet Earth^5^, while electric fields can induce curved growth of *Enterobacter cloacae* and *Escherichia coli* cells, which can rapidly curve towards the cathode in the post-division state, with a higher growth rate at the cell ends facing the anode than the ends facing the cathode^6^. The electrotaxis of Paramecium cells can be attributed to the depolarization of ionic species within the cell, leading to a torque orienting the body towards the cathode. A physical model was developed to describe their galvanotaxis by linking their microscopic ciliary motion with the macroscopic behavior^7^.

On the other hand, chemotaxis has been studied in physical chemistry systems as they can give an insight into how an artificial system can be used to represent or understand complex biological behaviors^8^. For example, chemotaxis of chemotactic droplets containing an organic solvent and 2-hexyldecanoic acid has been used to solve a constructed maze, where the motion of the droplets was driven by a pH gradient within the maze^9^. In a related work, Čejková et al.^10^ found that a gradient in salt concentration could drive the translational motion of alcohol droplets in an aqueous solution of sodium decanoate. Manipulation of the motion of a mercury droplet submerged in a conductive liquid medium by using a direct electric field was studied by Hollo et al.^11^. The electric potential drop across the droplet was found to generate Marangoni flow within the liquid which drives the droplet to move towards the cathode. The moving velocity of the droplet is proportional to the electric current density. They also showed that the droplet motion direction can be further manipulated by changing the conductivity of the liquid medium.

Kitahata et al.^12^ proposed a mechanism of hydrodynamics coupled with a chemical reaction to explain the translational motion of water droplets in an alcohol phase where the Marangoni effect is responsible for the droplet motion. In another work, the collective behavior of artificial squirmers was investigated for understanding large-scale biological pattern formation^13^.

Belousov-Zhabotinsky (BZ) reaction is a well-studied non-equilibrium reaction-diffusion process demonstrating unusual oscillatory properties^14,15^. It involves the oxidation of malonic acid to carbon dioxide by bromate ions in the presence of a transition metal catalyst, such as Ferroin solution. The BZ reaction can be described by the Field-Körös-Noyes (FKN) mechanism which consists of three competing processes and was theoretically described by the Oregonator model.^16,17^ Processes A and B form an inorganic subsystem of the BZ reaction and function as a so-called chemical clock. Process (A) acts as a long induction period that involves the consumption of Br$^{-}$ ions by multiple reactions. In Process B, increased production of HBrO$_3$ occurs followed by the oxidation of the ferroin solution. This is followed by Process C, which acts as the reset for the chemical clock. This reset causes the reduction of ferroin and bromide ions are produced in greater quantities again, which leads to the dominance of the reactions governing Process A. The type of process in which the solution alternates from the reduced state to the oxidized state is known as a redox reaction. The oxidation process can be visualized by the color change of the metal catalyst from red (reduced) to blue (oxidized). The BZ reaction produces target wave patterns in quasi-2D systems^18,19^, which have long been studied for their nonlinear dynamics^20,21^. For example, when an aqueous BZ solution is contained in a shallow dish, chemical waves are generated at locations called leading centers (LCs) and propagate towards the edge of the dish^22,23^. The formation of the LCs and subsequent wave propagation are typically of an unpredictable nature but can be manipulated by external interference, such as the application of an external electric field as studied in the current work.

Previous experimental works have shown that adjusting the concentration of metal catalysts, e.g., Ferroin can alter the propagating speed of the chemical wave through the BZ solution^24^. Such adjustment in catalyst concentration was also shown to impact the beating frequency, amplitude, and wave count, where the study of wave propagation along the arrays of contacted BZ droplets formed in the oil phase was conducted^25^. On the other hand, Steinbock et al.^26^ found that the oscillatory dynamics of BZ droplets depends on the size of the droplets.

Studies on the effect of external electric or magnetic fields on chemical wave generation and propagation in quasi-2D BZ solutions have led to the discovery of taxis behavior of BZ droplets. Agladze et al.^27^ showed that applying an electric field to gels containing BZ solutions can increase the propagation speed of the spiral waves within the gel. Blank and Soo^28,29^ reported that low-frequency electromagnetic fields can accelerate the BZ reaction, and consequently the wave travelling speed. It was also found that modulation of externally applied electric potential could alter the dynamic state of BZ microbeads from global oscillation to travelling waves or vice versa^30^. Furthermore, Okano et al.^31^ showed that the application of a static magnetic field can also increase the wave propagation speed in quasi-2D BZ solution.

Another interesting taxis phenomenon is the phototaxis of Ruthenium-catalysed droplets containing BZ solutions in the presence of a spatial gradient of light intensity^32,33^. These droplets undergo translational motion due to the phototactic effect, but no local switchback was observed. They can be even fixed in place when exposed to a light source right after the chemically driven translational motion^32^. The motion of the phototactic BZ droplets can be guided by using computer vision software to track and apply a spatial gradient of illumination to alter their moving direction. It was discovered that these droplets could follow a predetermined path guided by updating a control box each second^33^.

In this study, we investigated the electrotaxis behavior of BZ droplets with varied Ferroin concentrations suspended in an oil phase under the effect of electric fields of differing strength. We focus on analysing the field-induced inhomogeneous distribution of the leading center locations and the resulting translational motion of the droplets in parallel to the electric field. The magnitude of the electrotaxis effect was found to increase with the electric field strength. To the best of our knowledge, this is the first experimental demonstration of electrotaxis of BZ droplets. The physical mechanism of using an electric field to manipulate the direction of motion of self-propelled BZ droplets is of fundamental difference from the photochemical mechanism underlying the photo illumination method used to control the motion of photosensitive BZ droplets^32,33^. The electrotaxis of BZ droplets can find potential applications in biological environments and confined systems where a light source is hard to apply, and also call for new theoretical model descriptions.

## Results

As described in the “Experimental methods” section, the BZ droplets we studied are submerged in the oil phase contained in a petri dish. We tracked their trajectories individually and quantified their moving directions as well as the locations of the leading centers formed within the droplets. The bottom of the petri dish has a convex shape with the middle slightly thicker than the edges. The droplets thus have a trend of slowly drifting towards the edge under gravity, see Supplementary Information. Such drifting behavior is interrupted by the electrotaxis effect when a chemical wave is generated from the leading center formed inside a droplet and propagates across its body, which induces a translational motion of the droplet along the electric field direction. Once the chemical wave ceases, the drifting motion due to gravity resumes. Our analysis of the electrotaxis effect is thus focused on the trajectories within the time window where a chemical wave is generated and propagating across the body of the BZ droplet.

**Figure 1:**
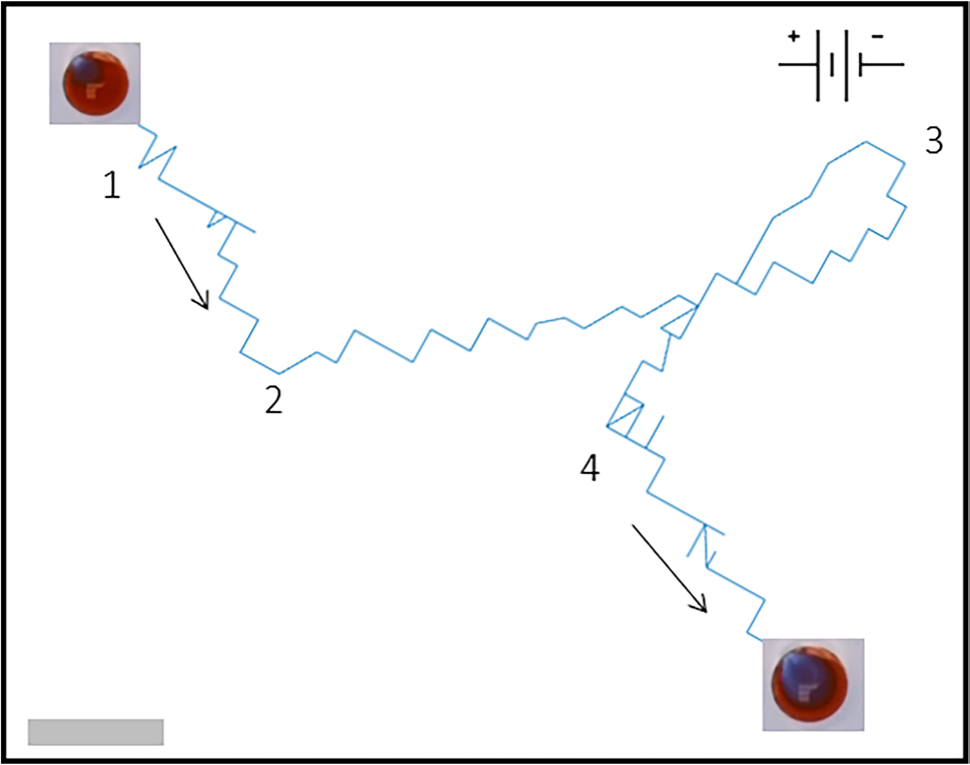
Trajectory of a BZ droplet moving under a DC electric field. The electric field was applied with direction horizontally from left to right, see the setup sketched in the top right corner. The droplet initially drifts along a path diagonally across the figure, as indicated by the black arrow with Label 1, but undergoes a chemically-driven translational motion from Point 2 to Point 3 in accordance with the direction of the electric field and also chemical wave propagation across the droplet body. After that, its trajectory shows a partial reset with the droplet moving back from Point 3 towards Point 2, but does not reach there. Due to the cessation of the chemical wave propagation, the droplet resumes its original drifting in the direction marked by the arrow with Label 4. The video clip recording this trajectory can be found in the SI. The scale bar at the lower-left corner represents 0.5 mm of the droplet trajectory.

One example moving trajectory of a BZ droplet under an external electric field is shown in Fig. 1. The DC field is applied using plate electrodes external to the droplet, represented with a sketch in the top right corner of the figure. The coordinate of the center of mass (CoM) of the droplet was extracted from the time-lapse images, as the droplet drifts across the oil phase. The initial drifting of the droplet in the circular petri dish with convex shaped bottom is roughly along the radial direction towards its edge. In Fig. 1, for convenience of demonstration, the coordinates of the experimental system have been rotated so that the electric field is directed horizontally from left to right. The initial drifting direction of the droplet turns out to be roughly along the diagonal line of the figure, as shown by the arrow with Label 1, which is interrupted when the chemical wave generated from a LC propagates across the droplet. This leads to the translational motion of the droplet in the direction of the applied field, see the trajectory from Point 2 to Point 3. After that, the trajectory shows a local switchback by moving back towards the onset point of the translational motion but does not fully return to Point 2. Due to inertia and hydrodynamic effects, the back-turn of the droplet does not happen sharply but goes through a small looping path about Point 3. After the wave propagation ceases, the droplet resumes its drifting behavior along the initial direction, as indicated by the arrow marked with Label 4. We note that the trajectory contains some small discrepancies in the droplet’s position due to subtle noise picked up when analysing the timelapse footage, which is represented by the aberrations away from pure vectorial motion.

**Figure 2:**
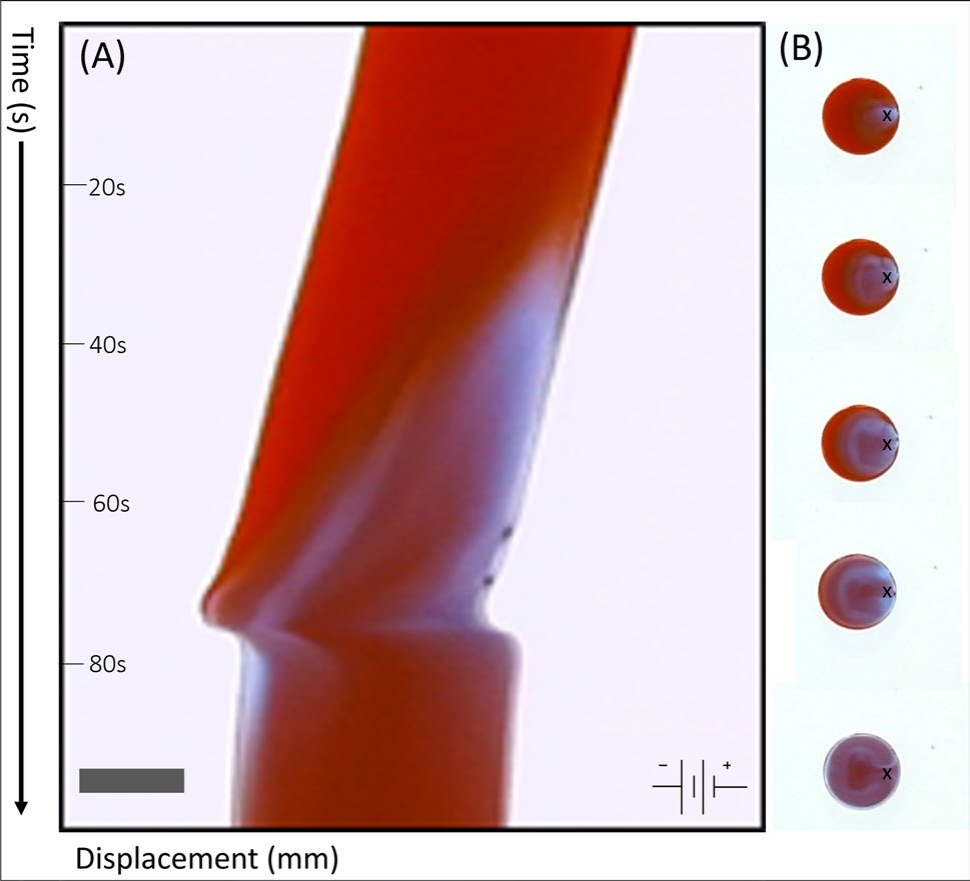
Motion of the droplet driven by the chemical wave. (**A**) Kymograph demonstrating the spatial and temporal pattern of chemical wave propagation in a BZ droplet. The electric field is in the horizontal direction pointing to the left. The scale bar represents 2 mm. The total time duration was 100 s. (**B**) Time-lapse images of the droplet at the sampling rate of 10 s. The location of the leading center is marked with an ’x’. The wave propagation can be seen from the color changes in the images.

Figure 2A shows the temporal and spatial propagation of the chemical wave across a single droplet in the presence of an electric field. This space-time image, known as a kymograph, was created by taking thin diametric segments coaxial to the electric field from the droplet images recorded at a frequency of 5 Hz over a 100 s window and stacking them downwards along the ordinate axis. The redox process and associated chemical wave propagation can be visualized from the color changes in the kymograph. The displacement of the droplet along the horizontal (electric field) direction can be deduced from the spatial location of the segment along the abscissa axis. Sampled images of the entire droplet taken as the sampling rate of 10 s are given in Fig. 2B. The motion of the droplet before and after the chemical wave propagation (and corresponding chemically driven translational motion) was the slow drifting towards the edge of the petri dish. This drifting is overcome by the chemically induced translational motion which alters the moving direction and trajectory of the droplet until the chemical wave ceases.

In connection with the droplet trajectory shown in Fig. 1, we can also see in Fig. 2A that the color of the entire segment remains roughly homogeneous and unchanged during the initial drifting process until a leading center appears at its right-hand-side (rhs), see also the droplet image on the top of Fig. 2B. The biased location of the LC under the external electric field will be discussed in more detail below. A wave of oxidized Ferroin is generated from the LC and propagates towards the other side of the droplet. When it approaches the droplet-oil interface, the droplet undergoes a fast translational motion, which is driven by the flow induced by the Marangoni stress generated by the gradient of interfacial tension between the two phases in response to the chemical wave.

**Figure 3:**
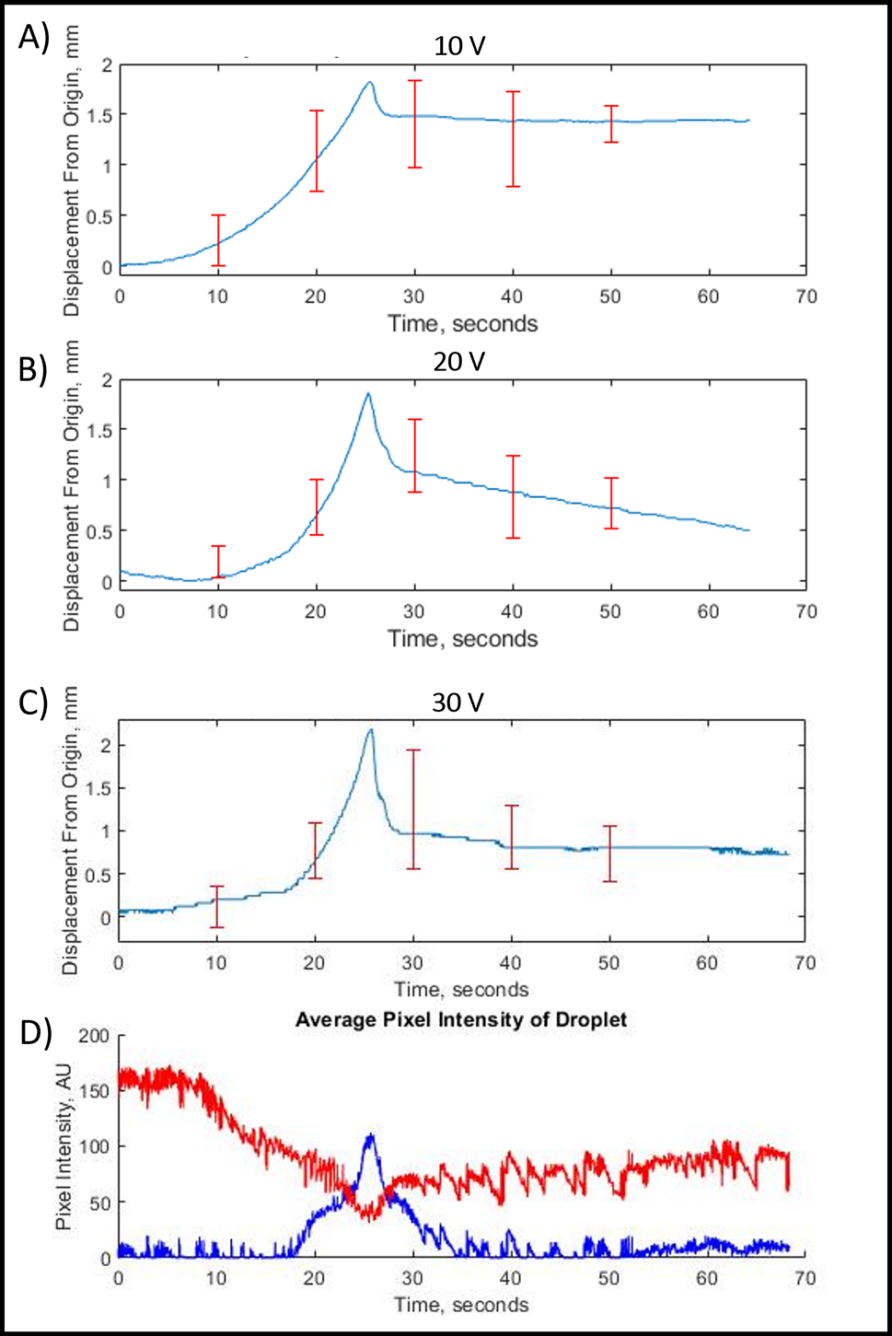
Relationship between the displacement of droplets and the redox states within droplets. (**A**–**C**) Average droplet displacement as a function of time from droplets subject to DC fields with 10 V, 20 V and 30 V, respectively. All curves were averaged over 10–15 sample data sets by shifting their peak displacements to the same reference time, and the error bars represent the standard deviations in the data sets. (**D**) Average pixel intensity values for the red and blue channels of the droplet RGB images. The chemical wave propagates across the droplet driving it to undergo translational motion under a 30 V DC field.

To quantify the chemically induced translational motion of the droplets, we extracted the center of mass of the droplets using image processing, and stored them in time series data sets. Figure 3A–C shows the average displacements of the droplets undergoing translational motion at DC field strengths 10 V, 20 V, and 30 V, respectively. In each case, the results were obtained by averaging over 10–15 independently measured data sets after shifting their peak positions to the same reference time point. The error bars reflect the standard deviations in the data sets calculated at 10 s intervals using statistical tests. For the convenience of comparison, the locations of the displacement peaks in Panels (A–C) are also aligned with each other. With the increase of the electric field strength, both the growth and decay rates of the displacement curves around their peaks, corresponding to the average velocities of the droplets moving along and against the electric field direction, show increments. For example, the average velocity moving along the field direction increases from 0.14 mm/s at 10 V to 0.28 mm/s at 30 V, and the average velocity moving against the field direction changes from − 0.23 mm/s at 10 V to − 0.48 mm/s at 30 V. The peak or maximum translational displacement also increases from 1.82 mm at 10 V to 2.1 mm at 30 V.

The decrease in the displacement value after the peak reflects the local switchback of the droplet position, which happens at a higher speed than the growth of the displacement. The non-zero plateau value at longer times indicates that the droplet does not fully return to its original position at the onset of the chemically-driven translational motion, in consistence with Figs. 1 and 2. The magnitude of the switchback displacement also shows a larger amplitude at higher field strength. The enhanced translational and switchback motions with increasing field strength can be related to the closer proximity of the lead centers formed at higher field strength to the edge of the droplets, as will be discussed below. We should note that the field-strength dependent effects observed in Fig. 3A–C are still within the statistical errors of our measurements. Further studies using a wider range of electric field strengths and larger numbers of samples are still needed to build up quantitative relationships between the velocity of translational motion as a function of the field strength.

A small sample of BZ solution undergoing a homogeneous redox process will change its color periodically from red (reduced state of Ferroin) to light blue (oxidized state of Ferroin). However, in the BZ droplet of millimeter size, the redox process and color transition do not occur homogeneously or instantaneously across the entire droplet. Instead, the chemical wave is generated from a LC where the oxidation of Ferroin begins and propagates within the droplet, see Fig. 2. The wave generation and propagation process can be quantified by the time-dependent red and blue pixel intensity values inside the droplets, as shown in Fig. 3D for the droplets studied in Fig. 3C. These data were collected by defining a $9 \times 9$ grid centered around the CoM of each droplet and averaging the pixel intensity values from the red and blue channels of their RGB images over the same time window as in Fig. 3C. The red pixel value decreases as the blue value increases, representing the oxidation process where the droplet’s color changes from red to blue. The maximum of the blue pixel intensity is reached when the chemical wave has propagated across the entire droplet and so the droplet is in the maximum oxidation state. The simultaneous occurrence of this oxidation peak value with the maximum droplet displacement shown in Fig. 3C supports the physical picture that the electrotaxis of BZ droplet is driven by the chemical wave propagation and the resulting gradient in the interfacial tension between the droplet and oil phases. The decay in the red values is found to occur prior to the growth in the blue values, and both the decay and growth processes of the red pixel values take much longer times to return to the pre-oscillation levels than the blue values. These results are consistent with the fast oxidation and slow reduction observed in BZ solutions. A lingering blue color remains for a short time after the translational motion has ceased.

The switchback of the moving trajectory takes place when the oxidized chemical wave starts to cease after reaching the droplet-oil interface on the opposite side of the droplet and the Ferroin begins to return to the reduced state, see Fig. 2. The changes in the wave propagation status and Ferroin redox state effectively reverse the gradient of the interfacial tension across the droplet, generating a momentum transfer in the opposite direction to the wave propagation direction and consequently, a reversed moving direction of the droplet^34,35^.

**Figure 4:**
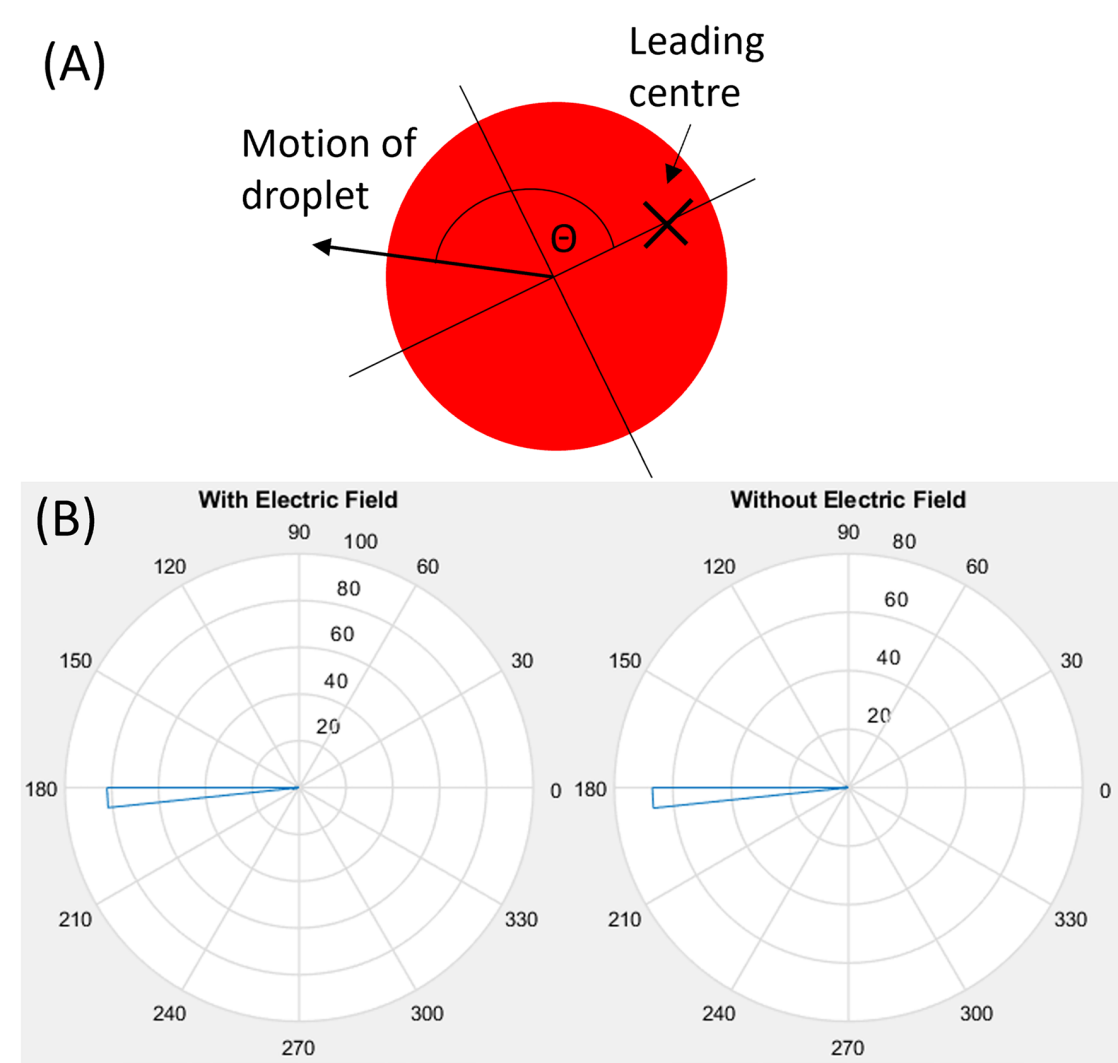
Travelling angle of the droplets in the coordinate system with respect to the leading centre. (**A**) Shematic of the 2D coordinate system defined on a droplet where the origin is at the CoM of the droplet and the x-axis pointing from the CoM to the location of the leading center. The angle $\theta$ is defined between the travelling direction of the droplet with respect to the x-axis. (**B**) Circular histogram of the travelling angle $\theta$ measured with and without an external electric field.

**Figure 5:**
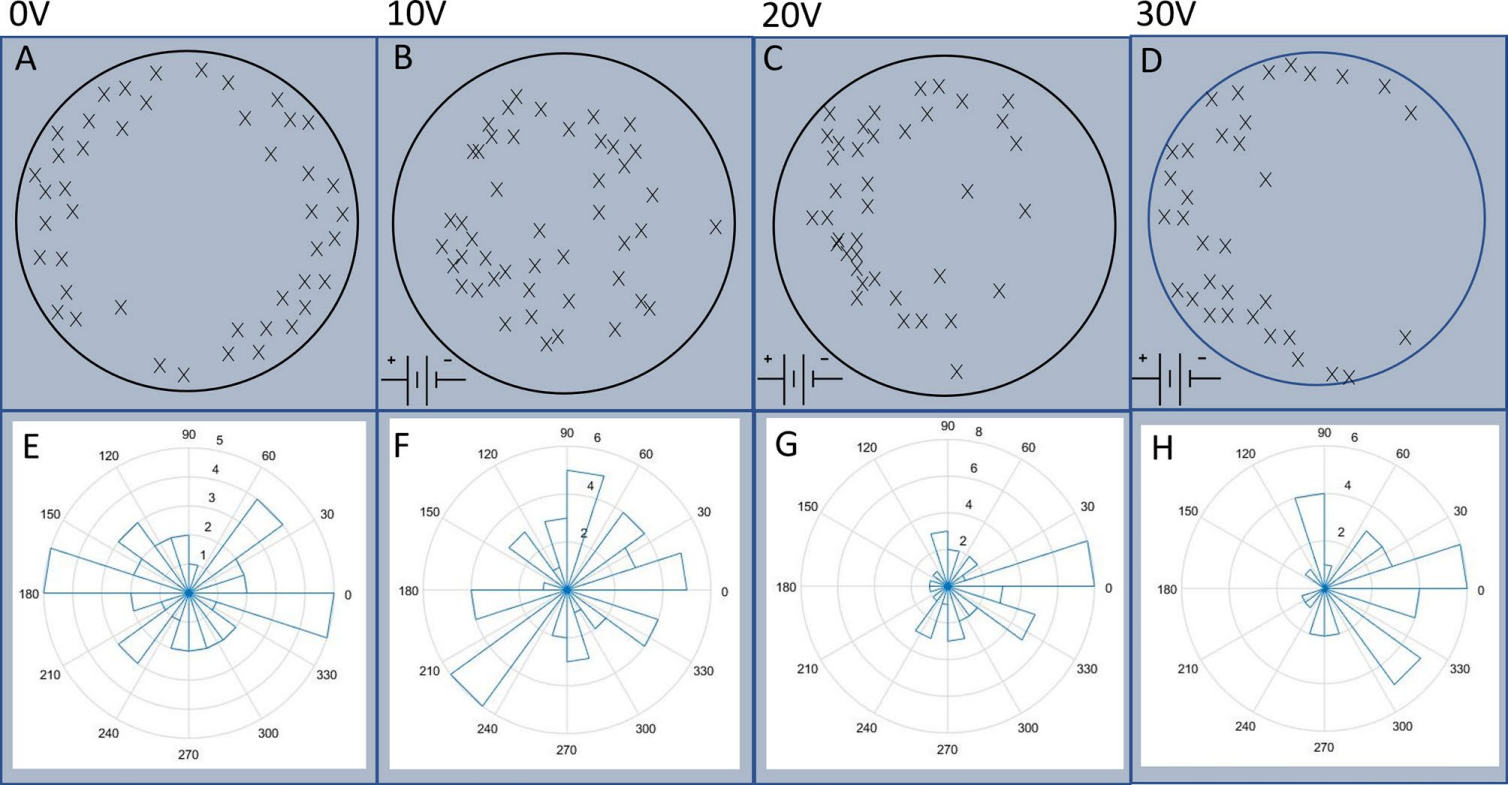
Relationship between the location of the leading centre and the travelling direction of droplets. (**A**–**D**) Distributions of the leading center locations (marked with x) within a schematic BZ droplet at different external electric field strengths. (**E**–**H**) Circular histogram of the travelling angles of chemically driven translation motion of droplet. The electric field was applied with the positive electrode on the left, and the negative electrode on the right. The applied voltages across the petri dish are shown in the labels on the top row. Data points were collected from 163 droplets.

In order to directly find the correlation between the location of the LC and the direction of the chemically driven translation motion of the droplet, we calculated the travelling angle $\theta$ of the droplet in the 2D coordinate system defined in Fig. 4A where the origin is at the CoM of the droplet and the x-axis is along the vector pointing from the CoM to the LC location, which is in the opposite direction of the wave propagation. Figure 4B presents the circular histogram of the angle $\theta$ between the translation motion direction and the x-axis in cases both with and without external field. The results show clearly that $\theta$ is centered around 180$^{\circ }$, indicating that the direction of the chemically driven translational motion is indeed determined by the location of the LC and pointing from the LC to the CoM of the droplet. This is well consistent with Fig. 5, i.e., the direction of translation motion is guided by the wave propagation from the LC.

Since the locations of the LCs determine the chemical wave generation/propagation process and consequently translational motion of the droplets, we carried out a systematic study of the effect of electric field strength on the spatial distributions of the LCs. Figure 5A–D show the results collected from 163 experiments and superimposed onto a model droplet at four different electric field strengths, i.e., $E=0, 1.1, 2.2$ and 3.3 V/cm. In these experiments, the concentration of Ferroin in the droplets is kept at 25 mM. In our testing experiments reported in the “Experimental method” section (see Fig. 8), a change of the Ferroin concentration from 15 to 35 mM makes no significant difference in the spatial distribution of the LCs both in the absence or presence of the external electric field. This implies that the electrotaxis of BZ droplets reported in this work is insensitive to the Ferroin concentration, as long as it is high enough to trigger the BZ reaction. So we will only present experimental results obtained at one Ferroin concentration (25 mM).

Figure 5 shows clearly that under zero or weak electric fields (A, B), the LCs are randomly formed inside the droplet, but at higher field strengths (C, D), the LCs have a strong tendency to be formed closer to the positive electrode. Chemical waves are generated from these LCs and propagate across the droplet bodies until reaching droplet-oil interfaces on both sides. Waves generated from the LCs located close to the droplet-oil interface can travel relatively long distances by passing the CoM of the droplet and reaching the interface on the opposite side. Such waves typically lead to larger translational displacement than those generated from the LCs close to the CoM of the droplets.

To quantify the effect of the electric field strength on the droplet motion direction, we use the coordinate system defined in Fig. 7 with the origin at the CoM of the droplet and the x-axis along the electric field direction. The travelling direction of the droplet translational motion can be conveniently defined by the tangential angle of the trajectory, $\tan ^{-1}(y/x)$, at the origin. The experimental results on the travelling angles are collected from the recorded trajectories and presented in Fig. 5E–H. Under zero or weak electric fields (E, F), the angles are in general uniformly distributed over 0$^{\circ }$–360$^{\circ }$, as expected from the unbiased distribution of the LC locations. However, the distribution narrows down to the two right quadrants ($0^{\circ }$–90$^{\circ }$ and $270^{\circ }$–360$^{\circ }$), because the LCs are dominantly formed on the left side of the droplet close to the positive electrode. These results clearly indicate that the chemically driven translational motion of the BZ droplets is in alignment with the electric field direction. Increasing the applied field strength will produce a greater taxis effect on the droplet.

**Figure 6:**
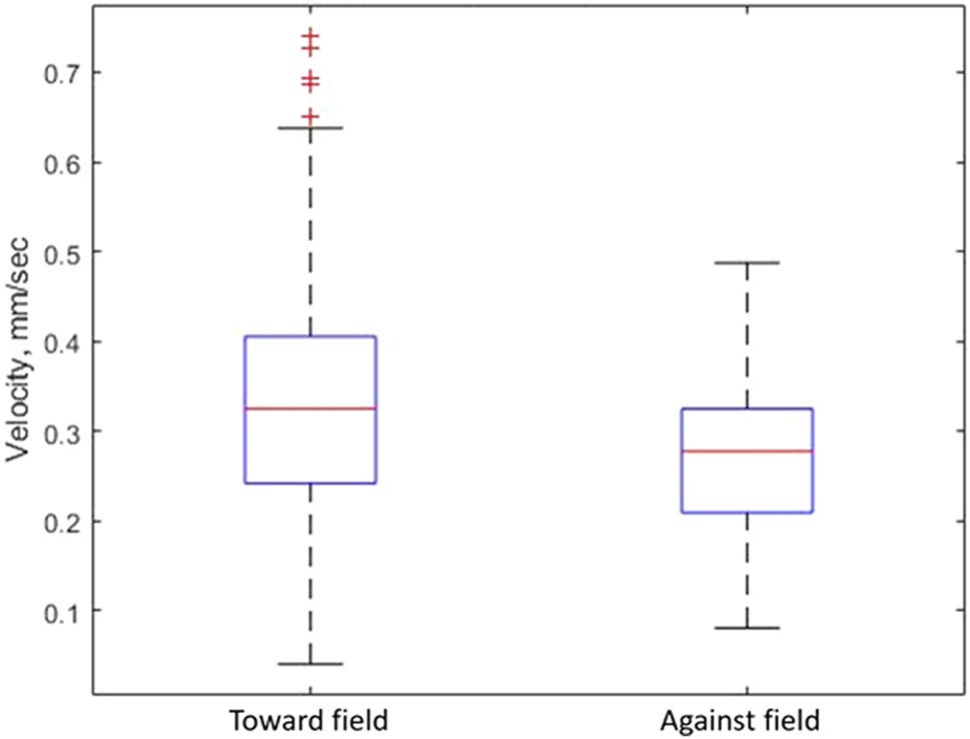
Boxplots showing propagation speeds of chemical waves. Speeds were compared travelling along and against the electric field direction. The median is shown with the red line, and the upper and lower quartiles are shown with the blue box. The black lines show the min and max values, and the red crosses show outliers.

For further information, we also measured the chemical wave propagation speed within the BZ droplets travelling towards and against the electric field. Figure 6 shows that the average propagation speed is slightly higher when the wave travels along the electric field than travelling against it. This effect may need to be taken into account, when developing theoretical models to describe the electrotaxis phenomenon of BZ droplets^27,28^.

We also note that droplets formed by solutions without Ferroin catalyst do not produce chemical wave patterns and show no chemically driven translational motion. Therefore no electrotaxis was observed in such systems. The presence of Ferroin is vital for triggering the BZ reaction and electrotaxis of the studied droplets, although the results are rather insensitive to the Ferroin concentration over the range we investigated (15–35 mM).

## Discussion

The spontaneous motion of BZ droplets floating on an oil phase has been previously studied by Kitahata et al.^34–36^. The propagation of the chemical wave was found to induce inhomogeneous interfacial tension, which in turn generates convective flow at the interface between the BZ solution and oil phases due to the Marangoni effect. The flow velocity differs in the fluids with different viscosities, leading to a momentum exchange between the two phases via interfacial friction. The droplet gains momentum, or propulsion force, in the opposite direction of the flow and undergoes spontaneous motion^34,35,37^. The translational motion of BZ droplets can be interpreted using the same mechanism as a result of chemo-mechanical energy transduction. The key finding of the current work is that the direction and magnitude of the translational motion of BZ droplets can be controlled by applying an external electric field with different strengths.

To understand the electrotaxis of BZ droplets, we recall the FKN mechanism which describes the BZ reaction as three periodically repeated processes^16,17^. Process A acts as a long induction period that involves the consumption of Br$^{-}$ ions by multiple reactions. Process B is the autocatalytic part with increased production of HBrO$_3$ and fast oxidation of metal ions (Ferroin in our case). This is followed by Process C, which acts as the reset for the chemical clock. This reset causes the reduction of Ferroin, and bromide ions are produced in greater quantities again which leads to the dominance of the reactions governing process A. The process in which the solution alternates from the reduced state to the oxidized state is known as a redox reaction. In the BZ reaction, the oxidation process is visually shown by the color change of the metal catalyst (Ferroin) from red to blue. This event leads to the propagation of the chemical wave across the solution, as the reaction becomes oxidized at surrounding regions, followed by a slower reduction of the catalyst. 

Different from typical experiments on quasi-2D BZ solutions and BZ droplets without external field where the location of the wave formation is unpredictable^22,23^, the application of electric field leads to the biased spatial formation of LCs towards the positive electrode. This bias can be attributed to the field-induced concentration gradient of Br$^{-}$ ions across the droplet which causes an instability of the chemical reaction^38,39^. The increased density of Br$^{-}$ in the proximity of the positive electrode allows for Process A to dominate the reaction process, leading to the oxidation of metal catalyst Ferroin in Process B. The chemical wave is then generated from an LC location biased towards the positive electrode^16^. Our experiments revealed a strong correlation between the biased distribution of the LC locations and the direction of the chemically driven translation motion of the BZ droplets. Greater electric field strength was found to produce a greater control of the electrotaxis effect of the BZ droplets.

It was also observed that the BZ droplets underwent a reverse motion after reaching their maximum chemically driven displacement, but never fully returned to the original position at the onset of the translational motion, see Fig. 3. This phenomenon, which we call the local switchback, can be explained by the longer time that the droplet spent in the oxidized state during the chemical wave propagation, where the translation displacement took place than that in the reduced state after the chemical wave ceased, where it moved backwards.

The wave propagation speed is shown in Fig. 6 to be higher when the wave travels along the electric field direction than against it. This can presumably be explained by the fact that the oxidized Ferroin ion of 3+ valence experiences an electrostatic force pointing along the electric field lines, which induces the motion along the field direction and so speeds up the associated wave propagation.

The discovery of electrotaxis phenomena in BZ droplets has an interesting application in the role of self-oscillating hydrogels, employing the use of BZ solutions, where electrotaxis can be utilised to break symmetry within the isotropic gel, causing the generation of a LC^40^. This will act as a form of control for the origin of the BZ reaction within the gel. Therefore controlling the chemo-mechanical coupling of the gel will be in a future study of the BZ electrotaxis phenomena. The electrotaxis of BZ droplets can also find potential applications in confined systems where it is possible to apply a light resource and for some photosensitive materials which may suffer light damage if using phototaxis effects^33^.

## Conclusion

We presented the electrotaxis of aqueous droplets of BZ solutions suspended on an oil phase. The application of an external DC electric field leads to a density gradient in the Br$^{-}$ ions within the droplet, and consequently biased location of the leading centers in favour of the positive electrode. A chemical wave of oxidized metal catalyst Ferroin is generated from the LC and propagates across the droplet by passing its center of mass. When reaching the droplet-oil interface on the other side (close to the negative electrode), the chemical wave creates inhomogeneous interfacial tension between the two phases and in turn a convective flow. This results in a momentum exchange between the BZ solution in the droplet and the oil phase, effectively producing a force which moves the droplet translationally in the direction of the electric field.

The droplet also undergoes a so-called local switchback by moving in the reverse direction after reaching the maximum translational displacement after the wave ceases and the droplet is in the reduced state. The electrotaxis effect increases with the increase of the electric field strength. This effect is however insensitive to the concentration of the metal catalyst Ferroin as long as it is high enough to trigger the BZ reaction.

## Experimental methods

The solutions were prepared by mixing 2 ml sulfuric acid (H$_{2}$SO$_{4}$) with 10 g sodium bromate (NaBrO$_{3}$) in 67 ml H$_{2}$O, to produce *Solution A*. 1 g malonic acid (CH$_{2}$(COOH)$_{2}$) was dissolved into 10 ml H$_{2}$O to produce *Solution B*. Then 1 g sodium bromide(NaBr) was dissolved in 10 ml H$_{2}$O to produce *Solution C*. All the lab-grade chemicals were bought from Sigma-Aldrich and Thermo-Fisher.

The reaction was initiated by mixing a solution containing 6 ml of *Solution A*, 1 ml of *Solution B* and 0.5 ml of *Solution C*. Upon mixing the three solutions, the mixture turned into a cloudy orange color due to Bromine gas being released. This solution was then left for a period of up to 5 minutes to allow for the re-absorption of the Bromine. Once the solution returned to a colorless state, 1 ml of Ferroin solution was added.

A single BZ droplet of volume in the range of 25–100 $\upmu$l was then pipetted onto the oil phase of oleic acid in a 90 mm diameter petri dish where its images and moving trajectories were recorded to investigate the chemically driven translational motion. We studied the electrotaxis effects of droplets with volumes 25 $\upmu$l, 50 $\upmu$l, 75 $\upmu$l and 100 $\upmu$l, respectively. For each droplet size, a total number of 60–100 droplets were generated and tested. In droplets of larger sizes (75 $\upmu$l and 100 $\upmu$l), there are typically multiple leading centers co-existing within their bodies which are formed at different locations and at different moments. The chemical waves originating from these LCs are out of phase by different degrees and thus interfere with each other. The integrated driving forces acting on these relatively large droplets did not lead to translational displacements as significant as those observed in the droplets of 50 $\upmu$l size over the electric field strength range studied in this work. On the other hand, the dimensions of the 25 $\upmu$l droplets are comparable to the wavelength of the BZ waves, leaving little space for wave propagation. These small droplets are thus not suitable for observing the electrotaxis effect. For these reasons, we have chosen to use the 50 $\upmu$l droplets in the current work.

The motion of the BZ droplet was recorded, using a Nikon D7500 camera recording at 60 frames per second for 30 min. The images were analysed to track the droplet positions over time. A pixel grouping algorithm was applied to detect individual droplets and track their moving trajectories between consecutive images. The LC locations within the droplet were also recorded. As a reference experiment, the translational motion of the droplets in absence of electric stimulation was recorded. This allows comparison between droplets under the electric field effect and droplets without the electric field effect. As the droplets continued to produce chemical waves from the same leading center, each data point collected was from a new droplet.

Initial experiments were conducted to determine the effect of the Ferroin concentration on the electrotaxis. These experiments were carried out by recording the chemically induced translational motion of droplets subjected to either a 30 V electric field application or no electric field application, with the concentration of Ferroin varying from 15 to 35 mM.

**Figure 7:**
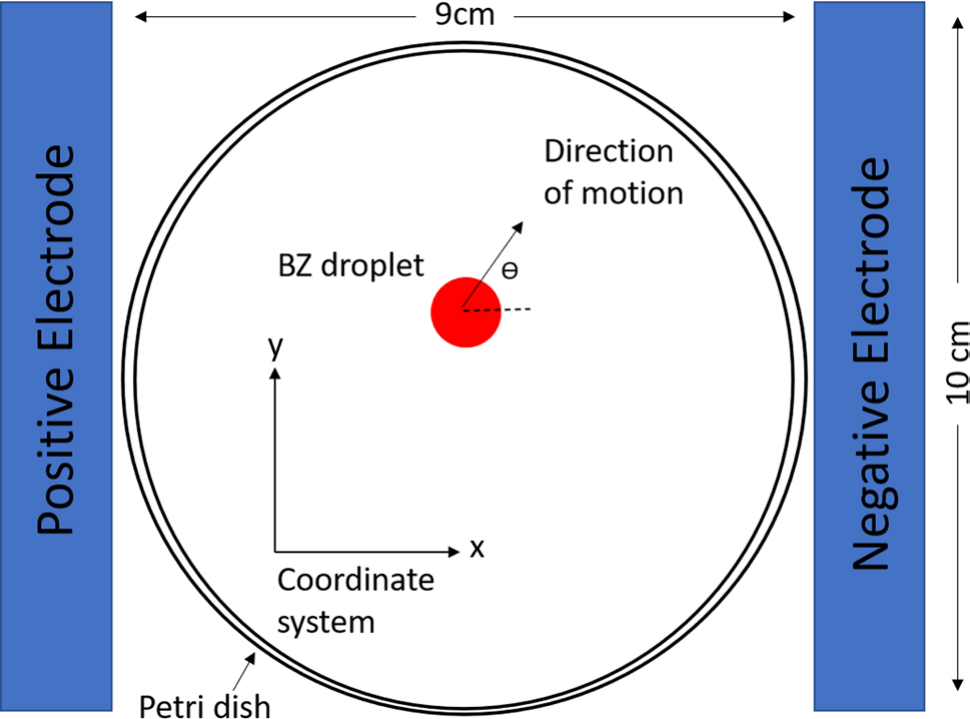
Schematics of the reaction vessel with plate electrodes. The *x*-*y* coordinate frame was set to calculate the travelling direction of the droplets. The red circle represents a moving BZ droplet, with an arrow showing its direction of translational motion. The angle $\theta$ shows the angle to the coaxial of the electric field in which the droplet travels. The electric field was applied in the strength range of 1.1–3.3 V/cm from the left to the right.

To apply the external electric field, a DC field (1.1–3.3 V/cm) was applied across the petri dish. The electric field was generated by placing a plate electrode at either end of the petri dish. Figure 7 shows the arrangement of the electrodes at each side of the reaction vessel, a petri dish with a diameter of 90 mm, with a voltage range of 10–30 V and a current of 3 A applied by a Tenma 72-10505 lab bench power supply. The electrodes were made of copper and have dimensions of 100 mm $\times$ 20 mm $\times$ 2 mm. For both sets of experiments, the droplets within the experimental system were recorded for 30 min at room temperature.

The visual images of the BZ droplets were taken to include the center of mass of the droplet. The color changes were analysed in the blue channel of an RGB image. To analyse the motion of the droplets, the *x*-*y* coordinate system was defined with the *y*-axis spanning the coaxial line of the electric field lines. The angle $\theta$, which measures the direction of the self-propelled droplets with respect to the *y*-axis, was calculated.

The location of the LC formation within the droplets was extracted and projected onto a circle representing the droplet (see Fig. 5) to show the distribution of the formations.

**Figure 8:**
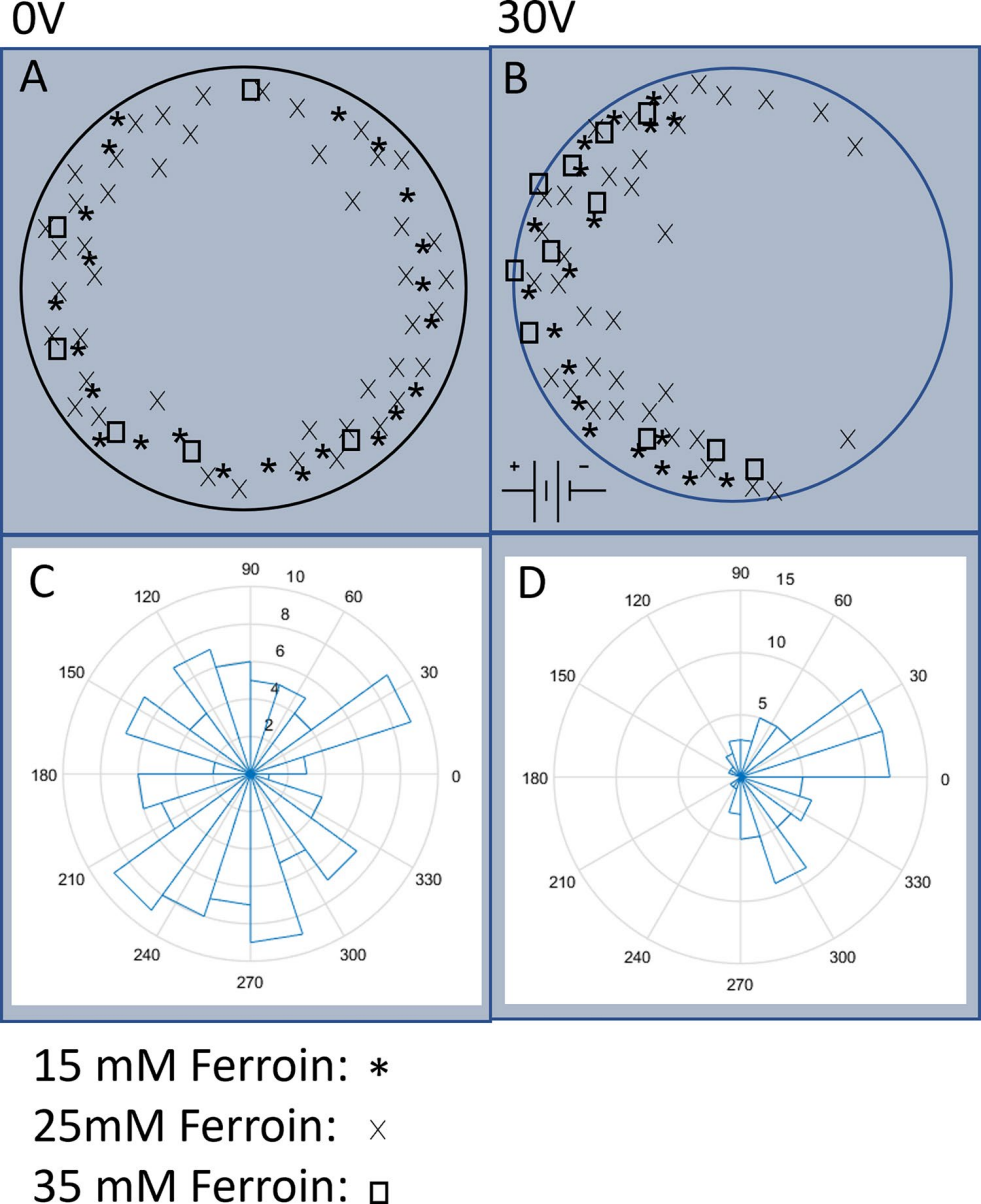
Effect of the Ferroin concentration on the electrotaxis of BZ droplets. (**A**, **B**) Distribution of the leading centers within an aqueous droplet under the application of an external DC electric field with strength 3.3V/cm, applied from left to right. (**B**), and for the control case with no external field applied (**A**). Experiment data obtained from droplets containing Ferroin concentrations of 15 mM, 25 mM and 35 mM are shown with an asterisk (*), cross (x) and open square symbols, respectively. (**C**, **D**) Circular histogram of the travelling angle of chemically driven translational motion of BZ droplets. The electric field was applied with the positive electrode on the left, and the negative electrode on the right. The field-induced biased distribution of LC formation in favour of the positive electrode is prominent when comparing (**B**) with (**A**). The data also shows that the taxis effect is insensitive to the Ferroin concentration. Data points were collected from 147 droplets.

Experiments have also been performed during a pilot study, to examine if the concentration of Ferroin has an effect on the electrotaxis of the BZ droplets. The data sets obtained using three different Ferroin concentrations (15, 25 and 35 mM) are presented in Fig. 8 and show no significant difference. We thus chose a Ferroin concentration of 25 mM for the remaining part of the study.

## Supplementary Information

Supplementary Information 1.

Supplementary Information 2.

Supplementary Information 3.

Supplementary Information 4.

Supplementary Information 5.

Supplementary Information 6.

Supplementary Information 7.

Supplementary Information 8.

Supplementary Information 9.

## Data Availability

Data is available upon request to the corresponding author.
