# Supplementary material for: Electrotaxis behavior of droplets composed of aqueous Belousov-Zhabotinsky solutions suspended in oil phase
Source: Sci Rep. 2023 Jan 24;13:1340. doi: 10.1038/s41598-023-27639-8 (PMC9873656; doi:10.1038/s41598-023-27639-8)
Supplement: Supplementary file 4 — Supplementary Information 4. [file 41598_2023_27639_MOESM4_ESM.docx]

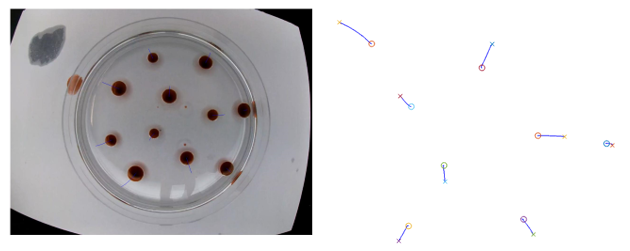


(Left) Snapshot of a petri dish containing a number of BZ droplets submerged in the oil phase, where the blue lines mark their drifting directions; (Right) Collection of drifting trajectories of BZ droplets with respect to the center of the petri dish. No electric field was applied in these experiments.
