# Supplementary material for: Electrotaxis behavior of droplets composed of aqueous Belousov-Zhabotinsky solutions suspended in oil phase
Source: Sci Rep. 2023 Jan 24;13:1340. doi: 10.1038/s41598-023-27639-8 (PMC9873656; doi:10.1038/s41598-023-27639-8)
Supplement: Supplementary file 5 — Supplementary Information 5. [file 41598_2023_27639_MOESM5_ESM.docx]

Drifting behavior was observed due to gravity acting on the droplets. The BZ droplets are suspended in an oil phase contained in a petri dish. As the middle portion is slightly thicker than the edges, a small incline is followed to the edges, and the droplets are shown to drift down as gravity acts on the droplets.

Link to find video with title same as this legend: <https://figshare.com/articles/media/Droplet_Drifting_Due_To_Gravity_MP4/21789182>
