# Supplementary material for: Electrotaxis behavior of droplets composed of aqueous Belousov-Zhabotinsky solutions suspended in oil phase
Source: Sci Rep. 2023 Jan 24;13:1340. doi: 10.1038/s41598-023-27639-8 (PMC9873656; doi:10.1038/s41598-023-27639-8)
Supplement: Supplementary file 6 — Supplementary Information 6. [file 41598_2023_27639_MOESM6_ESM.docx]

BZ droplets suspended in oil phase with no application of external DC field
