# Supplementary material for: Electrotaxis behavior of droplets composed of aqueous Belousov-Zhabotinsky solutions suspended in oil phase
Source: Sci Rep. 2023 Jan 24;13:1340. doi: 10.1038/s41598-023-27639-8 (PMC9873656; doi:10.1038/s41598-023-27639-8)
Supplement: Supplementary file 7 — Supplementary Information 7. [file 41598_2023_27639_MOESM7_ESM.docx]

In this supplemental video, a top-down view of BZ droplets is visible. The droplets are not subject to any external electric field.
