# Supplementary material for: Electrotaxis behavior of droplets composed of aqueous Belousov-Zhabotinsky solutions suspended in oil phase
Source: Sci Rep. 2023 Jan 24;13:1340. doi: 10.1038/s41598-023-27639-8 (PMC9873656; doi:10.1038/s41598-023-27639-8)
Supplement: Supplementary file 8 — Supplementary Information 8. [file 41598_2023_27639_MOESM8_ESM.docx]

TITLE: Aqueous droplet containing Belousov Zhabotinsky solutions undergoing chemically induced translational motion in direction of the externally applied electric field.

Legend: BZ droplet undergoing chemically induced translational motion
