# Supplementary material for: Electrotaxis behavior of droplets composed of aqueous Belousov-Zhabotinsky solutions suspended in oil phase
Source: Sci Rep. 2023 Jan 24;13:1340. doi: 10.1038/s41598-023-27639-8 (PMC9873656; doi:10.1038/s41598-023-27639-8)
Supplement: Supplementary file 9 — Supplementary Information 9. [file 41598_2023_27639_MOESM9_ESM.docx]

Supplemental video to figure 1.

Aqueous droplet containing BZ solutions undergoing chemically induced translational motion acting as an interrupt to the natural drifting motion observed in the droplet.
